# Accuracy of axillary ultrasound after different neoadjuvant chemotherapy cycles in breast cancer patients

**DOI:** 10.18632/oncotarget.13313

**Published:** 2016-11-11

**Authors:** Bei-Bei Ye, Hong-Meng Zhao, Yue Yu, Jie Ge, Xin Wang, Xu-Chen Cao

**Affiliations:** ^1^ The First Department of Breast Cancer, Tianjin Medical University Cancer Institute and Hospital, National Clinical Research Center for Cancer, Tianjin, China; ^2^ Key Laboratory of Cancer Prevention and Therapy, Tianjin, China; ^3^ Key Laboratory of Breast Cancer Prevention and Therapy, Tianjin Medical University, Ministry of Education, Tianjin, China

**Keywords:** breast cancer, neoadjuvant chemotherapy, axillary ultrasound, false-negative rate

## Abstract

**Background:**

This study determined whether axillary ultrasound (AUS) accurately predicted the status of axillary lymph nodes of patients who received different number of cycles of neoadjuvant chemotherapy (NAC).

**Materials and Methods:**

From 2008 to 2015, 656 cases of patients with breast cancers who received NAC and had subsequent axillary lymph node dissection were included in this study. The findings of preoperative AUS were tested by pathological examination. We evaluated the sensitivity, specificity and accuracy of AUS for patients who received two-, four-, and six-cycle NAC.

**Results:**

In the two-cycle subgroup, the sensitivity (Sn), specificity (Sp) and diagnostic odds ratio (DOR) were 80.2% (95% CI: 74.3%-86.2%), 61.4% (95% CI: 48.8%-74.0%) and 6.64 (95% CI: 3.36-12.4) respectively. In the four-cycle subgroup, the Sn, Sp and DOR were 69.7% (95% CI: 62.2%-77.1%), 66.1% (95% CI: 53.7%-78.5%) and 4.47 (95% CI: 2.32-8.62), respectively. In the six-cycle subgroup, the Sn, Sp and DOR were 56.7% (95% CI: 49.5%-64.0%), 74.5% (95% CI: 62.8%-87.2%) and 3.83 (95% CI: 1.863-7.86), respectively. Furthermore, the patients with normal AUS findings after six cycles of NAC have few positive nodes than patients with suspicious findings (*p* < 0.001).

**Conclusion:**

Preoperative AUS is a potentially useful imaging modality to predict the pathologic status of the axillary within four cycles of NAC. Although the accuracy is lower for patients who completed six cycles of NAC than that who received four- and two- cycles, the number of positive lymph nodes for patients with normal findings on AUS is low.

## INTRODUCTION

Breast cancer is one of the most frequent diagnosed cancers and the second most common cause of death in females around the world [1]. Neoadjuvant chemotherapy (NAC), which is known as primary or preoperative chemotherapy, is a treatment option for breast cancer patients with large primary tumors or locally advanced disease [2].NAC significantly contributes in controlling locoregional disease and improves the rate of breast conservation[3, 4]. A total of 23% to 32% patients with proven metastatic axillary lymph nodes exhibited no residual tumor cells in their lymph node after NAC [5, 6]. The residual of axillary lymph node disease after NAC is a prognostic marker for local recurrence and survival [7]. Axillary lymph node dissection (ALND) is the standard procedure for axillary lymph node staging and local control in breast cancer patients. Sentinel lymph node biopsy (SLNB) was also used as a method to obtain information on the status of axillary lymph nodes[8]. Current research aimed to identify novel and non-invasive nodal staging techniques with high sensitivity and negative predictive value (NPV) that could replace SLNB [9–13].

Although the optimal approach for the staging of axillary lymph nodes in patients receiving NAC remains unclear, axillary ultrasound (AUS) is commonly used in clinical practice in staging and following-up of regional lymph nodes. AUS has a sensitivity of 50%–70% and specificity of 87%–95% for breast cancer nodal metastasis [14–17]. The findings of abnormal nodes on AUS after NAC correlated with pathological residual positive nodes at surgery [18]. The Z1071 trial showed that 71.8% of patients who had suspicious nodes identified by AUS had residual node-positive disease after completing NAC [19]. The current study demonstrates the potential use of AUS after completing NAC before surgery to evaluate residual nodal disease. However, the accuracy of different number of cycles of NAC remains unclear. Therefore, the aim of this study was to evaluate the accuracy of AUS in detecting axillary lymph node metastases after different numbers of chemotherapy cycles.

## RESULTS

### Clinic–pathological features with the different chemotherapy cycle

We analyzed a total of 656 patients who received NAC and had subsequent ALND in our institute. The patients were divided into three groups based on the cycles of NAC: the two-cycle (*n* = 229), four-cycle (*n* = 201), and six-cycle (*n* = 226) subgroups. Table 1 describes the clinic–pathological features of the three groups. Age, menopausal status, clinical N stage, chemotherapy regimens, histological type, pathological tumor size, and pathological N stage, ER, HER2, Ki67, and the subtype of breast cancer were not significantly different among the three groups. The proportion of patients with T4 tumors at primary before NAC in the six-cycle subgroup (*p* < 0.001) was higher than that of patients in the two- or four-cycle subgroup. The patients in the four- or six-cycle subgroup achieved a more complete or partial response (CR/PR) to NAC and exhibited more significant shrinkage of tumors than patients in the two-cycle subgroup (*p* < 0.001). Comparison of the clinical–pathological features of the three groups indicates that the patients in six-cycle group have larger tumors at the primary but have better response to NAC than the other groups.

**Table 1:** Clinic-pathological features with the different chemotherapy cycle

Variables	Total (656)	2-cycle (229)	4-cycle (201)	6-cycle (226)	*P*-value
**Age(years)**					**0.158**
mean±SD	49.6± 9.8	50± 10.3	50± 9.49	49± 9.54	
**Menopausal status**					**0.69**
Pre-menopausal	346 (52.7%)	120 (52.4%)	102 (50.7%)	124 (54.9%)	
Post-menopausal	310 (47.3%)	109 (47.6%)	99 (49.3%)	102 (45.1%)	
**clinical T stage** **before NAC**					**<0.001**
cT1-2	386 (58.8%)	130 (56.8%)	133 (66.2%)	123 (54.4%)	
cT3	203 (30.9%)	83 (36.2%)	54 (26.9%)	66 (29.2%)	
cT4	67 (10.2%)	16 (7.0%)	14 (7.0%)	37 (16.4%)	
**clinical N stage** **before NAC**					**0.413**
cN0	118 (18.0%)	42 (18.3%)	41 (20.4%)	35 (15.5 %)	
≥cN1	538 (82.0%)	187 (81.7%)	160 (79.6%)	191 (84.5%)	
**clinical stage**					**0.211**
IIa-IIb	280 (42.7%)	99 (43.2%)	94 (46.8%)	87 (38.5%)	
IIIa-IIIc	376 (57.3%)	130 (56.8%)	107 (53.2%)	139 (61.5%)	
**Responseto chemotherapy**					**<0.001**
CR	41 (6.3%)	9 (3.9%)	15 (7.5%)	17 (7.5%)	
PR	323 (49.2%)	91 (39.7%)	108 (53.7%)	124 (54.9%)	
SD	261 (39.8%)	118 (51.5%)	70 (34.8%)	73 (32.3%)	
PD	31 (4.7%)	11 (4.8%)	8 (4.0%)	12 (5.3%)	
**Chemotherapy regimens**					**0.304**
T and (or) E regimens	606 (92.4%)	215 (93.9%)	181 (90.0%)	210 (92.9%)	
Others	50 (7.6%)	14 (6.1%)	20 (10.0%)	16 (7.1%)	
**Histological type**					**0.029**
IDC	544 (83.9%)	192 (83.8%)	174 (86.6%)	178 (78.8%)	
ILC	24 (3.7%)	11 (4.8%)	7 (3.5%)	6 (2.7%)	
Others	88 (13.4%)	26 (11.4%)	20 (10%)	42 (18.6%)	
**Estrogen receptor**					**0.189**
Negative	195 (29.7%)	74 (32.3%)	59 (29.4%)	62 (27.4%)	
Positive	461 (70.3%)	155 (67.7%)	142 (70.6%)	164 (72.6%)	
**Progesterone receptor**					**0.001**
Negative	317 (48.3%)	94 (41%)	91 (45.3%)	132 (58.4%)	
Positive	339 (51.7%)	135 (59%)	110 (54.7%)	94 (41.6%)	
**Her2/Neu**					**0.783**
Negative	561 (85.5%)	197 (86%)	169 (84.1%)	195 (86.3%)	
Positive	95 (14.5%)	32 (14.0%)	32 (15.9%)	31 (13.7%)	
**Ki-67 levels**					**0.082**
≤14%	198 (30.2%)	58 (25.3%)	61 (30.3%)	79 (35%)	
>14%	458 (69.8%)	171 (74.7%)	140 (69.7%)	147 (65%)	
**Breast cancer subtype**					**0.492**
Luminal A	152 (23.2%)	47 (20.5 %)	46 (22.9%)	59 (26.1%)	
Luminal B	326 (49.7%)	117 (51.1%)	103 (51.2%)	106 (46.9%)	
Her2-enriched	63 (9.6%)	20 (8.7%)	22 (10.9%)	21 (9.3%)	
TNBC	115 (17.5%)	45 (19.7%)	30 (14.9%)	40 (17.7%)	
**Pathological tumor size**					**0.062**
ypT0	53 (8.1%)	9 (3.9%)	19 (9.5%)	25 (11.1%)	
ypT1	185 (28.2%)	58 (25.3%)	55 (27.4%)	72 (31.9%)	
ypT2	311 (47.4%)	126 (55.0%)	96 (47.8%)	89 (39.4%)	
ypT3-T4	107 (16.3%)	36 (15.7%)	31 (15.4%)	40 (17.7%)	
**Pathological N stage**					**0.213**
pN0	155 (23.6%)	53 (23.1%)	56 (27.9%)	46 (20.4%)	
pN1	178 (27.1%)	59 (25.8%)	49 (24.4%)	70 (31.0%)	
pN2	149 (22.7%)	47 (20.5%)	51 (25.4%)	51 (22.6%)	
pN3	174 (26.5%)	70 (30.6%)	45 (22.4%)	59 (26.1%)	

NAC, neoadjuvant chemotherapy;TNBC,triple-negative breast cancer;

CR, complete response; PR, partial response;SD,stable disease;PD,progressive disease;

T, paclitaxel or docetaxel; E, anthracylines;

IDC, invasive ductal cancer;ILC, invasive lobular cancer.

### The accuracy of AUS after different cycles of NAC

Table 2 shows that the sensitivity of AUS in determining nodal involvement was 80.2% (95% CI: 74.3%-86.2%) in the two-cycle NAC group with a specificity of 61.4% (95% CI: 48.8%-74.0%). The accuracy was 75.5% (95% CI: 70.0%-81.1%). The positive likelihood ratio (+LR) was 2.07 (95% CI: 1.49-2.91), and the negative likelihood ratio (−LR) was 0.32 (95% CI: 0.22-0.46). The positive predictive value (PPV) and negative predictive value (NPV) were 86.3% (95% CI: 80.9%-91.6%) and 50.7% (95% CI: 38.9%-62.5%), respectively. The diagnostic odds ratio (DOR) was 6.46 (95% CI: 3.36-12.4). In the four-cycle group, the sensitivity was 69.7% (95% CI: 62.2%-77.1%), specificity was 66.1% (95% CI: 53.7%-78.5%), accuracy was 68.7% (95% CI: 62.2%-75.1%), +LR was 2.06 (95% CI: 1.40-3.00), -LR was 0.45 (95% CI: 0.34-0.63), PPV was 84.2% (95% CI: 77.64%-90.7%), NPV was 46.3% (95% CI: 34.8%-56.2%) and DOR was 4.47 (95% CI: 2.32-8.62). In the six-cycle group, the sensitivity was 56.7% (95% CI: 49.5%-64%), the specificity was 75.0% (95% CI: 62.8%-87.2%), accuracy was 60.6% (95% CI: 54.2%-67.0%), +LR was 2.22 (95% CI: 1.37-3.77), -LR was 0.58 (95% CI: 0.46-0.73), PPV was 89.4% (95% CI: 83.7%-95.1%), NPV was 31.3% (95% CI: 23.3%-40.4%) and DOR was 3.83 (95% CI: 1.863-7.86). As the number of cycles of chemotherapy increased, the sensitivity, DOR and NPV of AUS dramatically decreased and the false negative rate increased.

**Table 2:** The accuracy of AUS during different cycles of NAC

	2cycle (*n*= 229)	4cycle (*n*= 201)	6cycle (*n*= 226)	total (*n*= 656)
sensitivity (95%-CI)	80.2% (74.3%-86.2%)	69.7% (62.2%-77.1%)	56.7% (49.5%-64%)	68.7% (64.6%-72.8%)
specificity (95%-CI)	61.4% (48.8%-74.0%)	66.1% (53.7%-78.5%)	75.0% (62.8%-87.2%)	67.1% (59.8%-74.3%)
accuracy (95%-CI)	75.5% (70.0%-81.1%)	68.7% (62.2%-75.1%)	60.6% (54.2%-67.0%)	68.3 (64.7%-71.9%)
+LR (95%-CI)	2.07 (1.49-2.91)	2.06 (1.40-3.00)	2.22 (1.37-3.77)	2.08 (1.66-2.62)
-LR (95%-CI)	0.32 (0.22-0.46)	0.45 (0.34-0.63)	0.58 (0.46-0.73)	0.47 (0.39-0.55)
DOR (95%-CI)	6.46 (3.36-12.4)	4.47 (2.32-8.62)	3.83 (1.863-7.86)	4.43 (3.06-6.53)
PPV (95%-CI)	86.3% (80.9%-91.6%)	84.2% (77.64%-90.7%)	89.4% (83.7%-95.1%)	86.5% (83.1%-89.9%)
NPV (95%-CI)	50.7% (38.9%-62.5%)	46.3% (34.8%-56.2%)	31.3% (23.3%-40.4%)	40.8% (35.1%-47.0%)

+LR, positive linkhood ratios; -LR, negative linkhood ratios; DOR, diagnostic odds ratio;

PPV, positive predictive value; NPV, negative predictive value.

### False-negative AUS

Among the 656 patients, 262 patients exhibited normal findings in AUS after NAC. The 262 patients with negative AUS were examined. The patients were divided into two groups based on the pathological examination of the lymph nodes: the true-negative AUS group and the false-negative AUS group. Out of the 262 patients with negative AUS, 154 patients had a false-negative ultrasound (55.6%). These patients were found to have positive nodes after ALND. We compared the tumor characteristics between the true-negative and false-negative groups, and the results are summarized in Table 3. Age, histological type, menopausal status, nodal status, chemotherapy regimens, PR, HER2, were not significant in the two groups, which were excluded from further consideration. The clinical T stage (*p* < 0.01), cycles of chemotherapy (*p* = 0.018), pathological T stage (*p* < 0.001), ER status (*p* = 0.003), Ki67 (*p* = 0.034), and the subtype of breast cancer (*p* = 0.028) after NAC was significantly different between the two groups.

**Table 3:** Differences in patient and tumor characteristics between false-negative and true-negative axillary ultrasound

Variables	*N* (%) (262)	true negative (108)	false negative (154)	*P*-value
**Age(years)**				**0.916**
≤50	140(53.8%)	58(53.7%)	82(53.5%)	
>50	122(46.2%)	50(46.3%)	72(46.5%)	
**Menopausal status**				**0.383**
Pre-menopausal	148(56.5%)	58(53.7%)	90(58.5%)	
Post-menopausal	114(43.5%)	50(46.3%)	64(41.5%)	
**clinical T stage** **before NAC**				**<0.01**
cT1-T2	151(57.6%)	73(67.6%)	78(50.6%)	
cT3-T4	111(42.4%)	35(32.4%)	76(49.4%)	
**clinical N stage** **before NAC**				**0.099**
cN0	86(32.8%)	42(38.9%)	44(28.6%)	
≥cN1	176(67.2%)	66(61.1%)	110(71.4%)	
**Response to chemotherapy**				**0.141**
CR	29(11.1%)	15(13.9%)	14(9.1%)	
PR	146(55.7%)	65(60.2%)	81(52.6%)	
SD	77(29.4%)	26(24.1%)	51(33.1%)	
PD	10(3.8%)	2(1.9%)	8(5.2%)	
**Chemotherapy regimens**				**0.305**
Include T and (or) E regimens	242(92.4%)	102(94.5%)	140(90.9%)	
Others	20(7.6%)	6(5.5%)	14(9.1%)	
**The chemotherapy cycles before surgery**				**0.018**
2-cycle	69(26.3%)	35(32.4%)	34(22.1%)	
4-cycle	81(30.9%)	38(35.2%)	43(27.9%)	
6-cycle	112(42.7%)	35(32.4%)	77(50.0%)	
**Histological type**				**0.714**
IDC	169(64.5%)	69(63.9%)	100(64.9%)	
Others	93(35.5%)	39(36.1%)	54(35.1%)	
**Estrogen receptor**				**0.003**
Negative	72(27.5%)	40(37.0%)	32(20.8%)	
Positive	190(72.5%)	68(63.0%)	122(79.2%)	
**Progesterone receptor**				**0.685**
Negative	130(47.8%)	55(50.9%)	75(48.7%)	
Positive	132(52.2%)	53(49.1%)	79(51.3%)	
**Her2/Neu**				**0.117**
Negative	225(86.1%)	88(81.5%)	137(89.0%)	
Positive	37(13.9%)	20(18.5%)	17(11.0%)	
**Ki-67 levels**				**0.034**
≤14%	84(32.1%)	27(25.0%)	57(37.0%)	
>14%	178(67.9%)	81(75.0%)	97(63.0%)	
**Breast cancer subtype**				**0.028**
Luminal A	66(25.2%)	20(13.0%)	46(30.0%)	
Luminal B	131(50.0%)	53(49.1%)	78(50.6%)	
Her2-enriched	22(8.4%)	9(8.3%)	13(8.4%)	
TNBC	43(16.4%)	26(24.1%)	17(11.0%)	
**Pathological tumor size**				**<0.001**
ypT0	32(12.2%)	18(16.7%)	14(9.1%)	
ypT1	84(32.1%)	42(38.9%)	42(27.3%)	
ypT2	112(42.7%)	45(41.7%)	67(43.5%)	
ypT3-T4	34(13.0%)	3(2.8%)	31(20.1%)	

Univariate and multivariate analyses are summarized in Table 4. Both the univariate and multivariate analyses revealed that high number of cycles of NAC (*p* = 0.008) and large tumor size (*p* = 0.009) were associated with having a false-negative AUS for patients with normal AUS findings after NAC. Compared with the two-cycle group, patients with normal AUS findings after six cycles of NAC were more likely to find lymph nodes with residual tumor cells in pathologic examination. However, no significant difference was observed between the two-cycle group and the four-cycle group.

**Table 4:** Univariate and multivariate associations between tumor characteristics and false-negative axillary ultrasound

	Univariate analysis			Multivariate analysis		
Characteristics	HR	95%CI	*P*-value	HR	95%CI	*P*-value
**Primary Clinical T stage before NAC**						
T3 and T4 vs T1 and T2	2.032	1.218-3.391	0.007	1.509	0.855-2.663	0.155
**Number of NAC**						
4-cycle vs 2-cycle	1.224	0.643-2.329	0.538	1.147	0.580-2.267	0.694
6-cycle vs 2-cycle	2.173	1.17-4.025	0.014	2.505	1.270-4.941	0.008
**Pathological tumor size**						
>2cm vs ≤2cm	2.107	1.276-3.479	0.004	2.160	1.217-3.834	0.009
**Estrogen receptor**						
positive vs negative	2.334	1.34-4.06	0.003	5.000	0.839-29.804	0.077
**Ki-67 levels**						
>14% vs ≤14%	0.552	0.32-0.951	0.032	0.755	0.27-2.115	0.593
**Breast cancer subtype**						
Luminal B vs Luminal A	0.64	0.341-1.202	0.165	0.765	0.239-2.453	0.652
Her2-enriched vs Luminal A	0.628	0.231-1.705	0.361	3.751	0.442-31.858	0.226
TNBC vs Luminal A	0.284	0.127-0.636	0.002	1.643	0.212-12.744	0.635

### Association of AUS findings with pathologic findings for six-cycle subgroup

Of the 112 patients with normal findings assessed by AUS in the six-cycle group, 33 patients (29.5%, 95% CI: 21.0%-37.9%) was pN0, 38 patients (33.9%, 95% CI: 25.2%-42.7%) was pN1, 41 patients (36.6%, 95% CI: 27.7%-45.5%) have more than three metastasized nodes. In comparison, 113 patients who had suspicious nodes identified by AUS, 12 patients (10.6%, 95% CI: 4.9%-16.3%) was pN0, 32 patients (28.3%, 95% CI: 20.0%-36.6%) was pN1, 69 patients (61.1%, 95% CI: 52.1%-70.1%) were found to have more than three metastasized nodes. The patients with normal lymph nodes on AUS examinations after six cycles of NAC have few positive nodes than patients with suspicious findings (*p* < 0.001).

## DISCUSSION

Our data suggests that as the number of cycles of chemotherapy increased, the sensitivity, DOR and NPV of AUS dramatically decreased and the false negative rate increased. For patients exhibited normal findings in AUS after NAC, high number of cycles of NAC and large tumor size were associated with having a false-negative AUS. The patients with normal lymph nodes on AUS examinations after six cycles of NAC have a lower nodal burden than patients with suspicious findings (*p* < 0.001).

The Z0011 trial determined that dissecting the axillary in the presence of one to two positive SLN is not beneficial, which indicates that conducting an extensive surgery in the axillary does not improve the outcome [20]. We aim to identify a novel and non-invasive nodal staging technique with high sensitivity and NPV that could replace SLNB. The diagnostic performance of magnetic resonance imaging (MRI) in assessing axillary nodal staging and the response to NAC in breast cancer patients is promising because its NPV approaches the NPV of SLNB [21]. However, the cost of MRI is high, and it is not always available. Thus, this approach is not easily accepted by patients. AUS is non-invasive, widely available, low-cost, and commonly used in clinical practice. The SOUND trial was designed to determine whether surgical staging method could be replaced with an imaging method of staging the axillary that can diagnose relevant nodal involvement and replace SLNB [22]. Patients with either negative cytology of a single suspicious lymph node or with negative ultrasound of the axillary will be randomly eligible to undergo SLNB±axillary dissection group or no axillary surgical group. This trial is still ongoing, and the results are not yet reported [22].

Our results suggest that AUS is a useful imaging modality to assess the axillary response to chemotherapy regimens and the status of axillary lymph node for patients with four cycles of NAC. In addition, the sensitivity of AUS with two cycles of NAC reached 80.2%, which is as high as that of MRI [21]. However, when six cycles of NAC were adopted, the sensitivity decreased from 80.2% to 56.7%. After patients completed six cycles of NAC, the sensitivity of AUS decreased and the false-negative rate (FNR) reached 43.3%, which means that almost one in two persons with normal findings in AUS will be misdiagnosed. The confirmation whether the high FNR in this subgroup can triage patients for SLNB or evaluate the status of axillary lymph node accurately is unclear. Although the FNR is relative high for patients who completed six cycles of NAC, the overall nodal burden of disease for patients with normal findings on AUS is low with a few positive nodes (< four positive nodes in 75.3% of patients with normal lymph nodes). Basing on the results of Z1071 study, patients with normal lymph nodes in the AUS examination have a significantly lower likelihood of having residual disease[19]. Moreover, the normalized lymph node morphology on ultrasound after NAC was correlated with high pathological complete response (pCR) rates [23]. Thus, despite the high FNR of AUS, clinicians can still employ ultrasound to estimate the status of the axillary lymph node after six cycles of neoadjuvant treatment. However, another imaging modality with better sensitivity and specificity, such as positron emission tomography with computed tomography (PET/CT) or MRI, should be combined with AUS to identify patients who have clinically negative axillary after complete neoadjuvant treatment and who want to undergo axillary conservation surgery after completing six cycles of NAC [24–27].

M. Moormanet al. noted that young patients with large tumors and had lymph vascular invasion were more likely to exhibit lymph nodes with residual tumor cells in pathologic examination when the AUS were normal prior to surgery[28]. The results of univariate and multivariate analyses about the false-negative AUS in this work suggest that completing six cycles of NAC is probably one of the factors that affect the prognosis of patients. The treatment effect in lymph nodes may result in the fibrosis and apoptosis of tumor cells, which may either cause ambiguous morphologic changes on imaging or not. AUS that depicts lymph nodes with metastatic disease is based on morphologic criteria and size. Thus, changes in size and morphologic causes difficulties in identifying suspicious lymph nodes. Although completing six cycles of NAC obtained a better response, the axillary pathologic complete response (pCR) was not high. A study from the Netherlands suggests that only 20% of the patients with metastasis prior to NAC achieved an axillary pCR[29]. The false-negative AUS was high in tumors ≥ 2cm after neoadjuvant treatment. Large tumor size may indicate high tumor burden. A false-negative ultrasound more likely occurs in patients with large tumors because of the high pretest probability of metastatic disease; thus, a significant number of small axillary metastases may be missed[30]. Patients with the pCR of metastatic lymph nodes were reported to be more likely to have better prognosis despite having residual primary tumors[31]. The risk of local recurrence increases with the number of residual positive nodes after NAC[32]. Previous studies suggest that radiologic complete response has similar survival outcomes and recurrence-free survival to patients with pCR[33]. Our data shows that AUS is useful to evaluate the status of axillary lymph nodes with good sensitivity and specificity after NAC. However, further analysis is status necessary to determine whether AUS can replace SLNB in obtaining information on the status of axillary lymph nodes and identify patients with positive nodes as candidates for extensive surgical procedures. Limitations exist our study. Not all patients consented to the completion of ALND or some patients underwent AUS examination before surgery, thus could not be included in the analysis and decreasing our evaluable cohort. The criterion used for patients to receive NAC differs from others with a cortical thickness>3 mm with or without palpable axillary lymph node. Therefore, the criteria used for analyzing suspicious nodes mainly depend on the change of morphology in our institute.

## CONCLUSIONS

Our data indicate that preoperative AUS is a potentially useful imaging modality to predict the pathologic status of the axillary with four cycles of NAC, whereas the FNR is very high for patients who received six cycles. Although the accuracy for patients who completed six cycles of NAC is significantly lower than those who underwent four cycles of NAC, the overall nodal burden of disease for patients with normal findings on AUS is low. Clinicians can still choose to employ ultrasound to estimate the status of axillary lymph node after six cycles of neoadjuvant treatment. However, radiologists should focus on evaluating the status of axillary lymph nodes for patients with large tumors and those who completed six cycles of NAC. Another imaging modality with better sensitivity and specificity, such as PET/CT or MRI, should be combined with AUS for patients who want to undergo axillary conservation surgery after completing six cycles of NAC.

## PATIENTS AND METHODS

### Patient population

**Figure 1:**
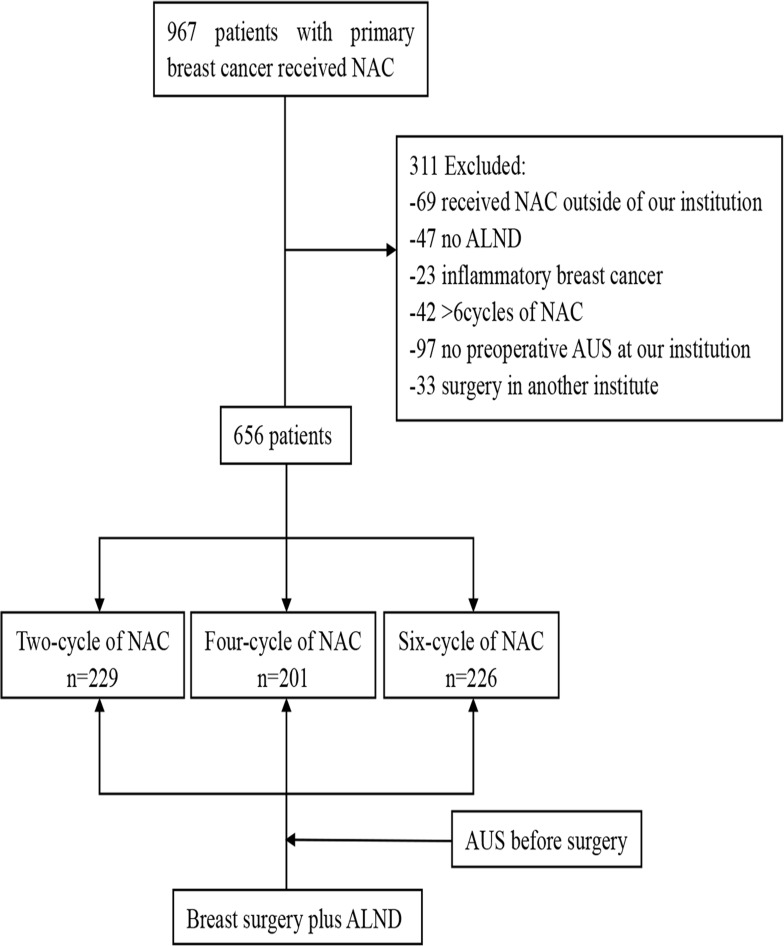
Study Flow chart

From January 2008 to October 2015, we retrospectively analyzed 967 consecutive breast cancer patients who received NAC and 656 patients were selected in this retrospective study. 311 patients were excluded from our study, including 69 patients whose NAC received outside of our institution, 23 patients with inflammatory breast cancer, 97 patients have no preoperative AUS, 47 patients not underwent ALND, 33 patients surgery in another institute and 42 patients received more than six cycles of NAC (Figure 1). The indications for NAC were primary tumors greater than 3 cm and/or with axillary lymph node metastasis and no evidence of distant metastases. All the patients included in the study met the following inclusion criteria: (1) All patients had histologically proven invasive carcinoma by core biopsy prior to commencing primary chemotherapy. (2)No patient had detectable metastatic disease before primary chemotherapy was instituted.(3)Preoperative AUS was performed in each patient.(4) All the patients underwent NAC and breast surgery plus axillary lymph node dissection (Level I and Level II)at our institution.(5) At least 15 lymph nodes were examined for pathological diagnosis. (6) None of the patients previously received neoadjuvant therapy. Informed consent was obtained from all the patients above and research protocol for this study was approved by the Ethics Committees at the Tianjin Medical University Cancer Institute and Hospital.

### Ultrasound imaging protocol

The patients underwent both the breast and AUS for initial staging at presentation using the ultrasound machine Legiq E9 (General Electric Co., USA) with 6-15 MHz linear transducer in our institute. Two experienced radiologists independently analyzed the images according to the classification of the American College of Radiology BI-RADS system (2003). Axillary lymph nodes were assessed by their shape and the morphology of the cortex. A lymph node was classified as suspicious if its cortical thickness was >3 mm or if it had an irregular nodular cortex and/or a diminished or absent hilum. Since the large number of patients who are waiting in line to underwent the core biopsy test, most patients with tumors greater than 3 cm gave up the test for suspicious metastasis lymph nodes and received NAC directly. Only a few patients with tumors less than 3 cm underwent core biopsy for suspicious metastasis lymph nodes. AUS was repeated before surgery.

### NAC and clinic-pathologic features

Most of the patients received anthracycline with or without taxane-based (epirubicin, cyclophosphamide, and paclitaxel, or epirubicin and paclitaxel) chemotherapy every three weeks for at least two cycles. The regimen and cycles of NAC were under the discretion of the treating medical oncologist. The responses of tumors to NAC were evaluated by RECIST 1.1 criteria [34]. All the criteria were applied in measuring the primary tumor only. Nodal metastases were not considered.

All the pathological specimens of the patients before and after NAC were microscopically reviewed. Histological type and biomarkers were diagnosed using specimens obtained after the modified radical mastectomy. Immunohistochemical staining for estrogen receptor (ER) and progesterone receptor (PR), as well as HER2, was performed. ER and PR were considered positive if nuclei stained>1% [35]. HER2 status was assessed by scoring the intensity of membrane staining using immunohistochemistry. Tumors with a score of 3+ (strong homogeneous staining) or with a >2.2-fold increase in HER2 gene amplification, which was determined by fluorescence in situ hybridization, were considered HER2-positive. Molecular subtypes were categorized into four subgroups as follows: luminal A, ER positive and/or PR positive, HER2 negative, and Ki-67≤14%; luminal B, ER positive and/or PR positive, HER2 positive and/or Ki-67 > 14%; HER2-enriched, ER negative, PR negative and HER2 3+; triple-negative breast cancer (TNBC), ER-negative, PR-negative and HER2-negative.

### Statistical analysis

The differences in the characteristics between patients who received different cycles of NAC from the analysis were tested using the t-statistic or F-statistic for continuous variable and χ^2^statistics or Kruskal-Wallis H test for categorical variables. Sensitivity (the proportion of patients with ypN+ who had a positive pre-op AUS) and specificity (the proportion of patients with ypN- who had a negative pre-op AUS) were calculated for AUS with the final pathologic findings as gold standard. The method that uses AUS was assessed by determining the positive and negative likelihood ratios. Multivariate logistic regression was used to determine the factors associated with a false-negative event. All the statistical tests were two-sided, and *P* < 0.05 was considered significant. SPSS software version 17.0 was utilized for the analysis.
